# Deciphering the Crucial Roles of the Quorum-Sensing Transcription Factor SdiA in NADPH Metabolism and (*S*)-Equol Production in *Escherichia coli* Nissle 1917

**DOI:** 10.3390/antiox13030259

**Published:** 2024-02-20

**Authors:** Zhe Wang, Yiqiang Dai, Fidelis Azi, Mingsheng Dong, Xiudong Xia

**Affiliations:** 1 College of Food Science and Technology, Nanjing Agricultural University, Nanjing 210095, China; wangzhe@stu.njau.edu.cn (Z.W.);; 2 Institute of Agro-Product Processing, Jiangsu Academy of Agricultural Sciences, Nanjing 210014, China; 3 Department of Chemical Engineering, Guangdong Technion-Israel Institute of Technology, Shantou 515063, China; 4 Jiangsu Key Laboratory for Food Quality and Safety-State Key Laboratory Cultivation Base, Ministry of Science and Technology, Nanjing 210014, China; 5 School of Food and Biological Engineering, Jiangsu University, Zhenjiang 212013, China

**Keywords:** isoflavone, (*S*)-equol, quorum sensing, *E. coli* Nissle 1917, NADPH, biosynthesis

## Abstract

The active metabolite (*S*)-equol, derived from daidzein by gut microbiota, exhibits superior antioxidative activity compared with its precursor and plays a vital role in human health. As only 25% to 50% of individuals can naturally produce equol when supplied with isoflavone, we engineered probiotic *E. coli* Nissle 1917 (EcN) to convert dietary isoflavones into (*S*)-equol, thus offering a strategy to mimic the gut phenotype of natural (*S*)-equol producers. However, co-fermentation of EcN-eq with fecal bacteria revealed that gut microbial metabolites decreased NADPH levels, hindering (*S*)-equol production. Transcriptome analysis showed that the quorum-sensing (QS) transcription factor SdiA negatively regulates NADPH levels and (*S*)-equol biosynthesis in EcN-eq. Screening AHLs showed that SdiA binding to C10-HSL negatively regulates the pentose phosphate pathway, reducing intracellular NADPH levels in EcN-eq. Molecular docking and dynamics simulations investigated the structural disparities in complexes formed by C10-HSL with SdiA from EcN or *E. coli* K12. Substituting *sdiA_EcN* in EcN-eq with *sdiA_K12* increased the intracellular NADPH/NADP^+^ ratio, enhancing (*S*)-equol production by 47%. These findings elucidate the impact of AHL-QS in the gut microbiota on EcN NADPH metabolism, offering insights for developing (*S*)-equol-producing EcN probiotics tailored to the gut environment.

## 1. Introduction

(*S*)-equol is a gut microbial metabolite of daidzein and daidzin, which are the major isoflavones in soybean, and it exhibits heightened antioxidative and estrogenic activities compared with its precursors [1,2]. Its potential in alleviating oxidative stress, menopausal syndrome, cardiovascular disease, and osteoporosis, as well as reducing the risk of prostate, colon, and breast cancer, has garnered substantial clinical interest [2,3]. However, only a fraction (25 to 50%) of people harbor gut bacteria with an (*S*)-equol-producing pathway capable of metabolizing dietary daidzein into (*S*)-equol [4,5]. Fecal transplant therapy has demonstrated success in conferring an (*S*)-equol-producing phenotype in hosts by providing them with exogenous gut bacteria expressing the necessary enzymes for daidzein conversion into (*S*)-equol [6,7]. Recently, engineered bacteria-integrated (*S*)-equol-producing pathways have been utilized as efficient whole-cell biocatalysts for (*S*)-equol production [3,8]. Given that EcN is a probiotic widely used as a host for the development of engineered probiotics [9,10], it can be used to develop an engineered probiotic with the capability of converting dietary isoflavones into (*S*)-equol efficiently [11].

The (*S*)-equol-producing pathway involves four enzymatic steps: daidzein reductase (DZNR) catalyzes the conversion of daidzein to (*R*)-dihydrodaidzein, which can further undergo conversion to (*S*)-dihydrodaidzein facilitated by dihydrodaidzein racemase (DDRC) [3,4]. The subsequent reduction of (*S*)-dihydrodaidzein to *t*-tetrahydrodaidzein occurs through dihydrodaidzein reductase (DHDR), followed by the transformation of *t*-tetrahydrodaidzein into (*S*)-equol via tetrahydrodaidzein reductase (THDR) [5]. Notably, the enzymes DZNR and DHDR belong to the category of NADPH-dependent oxidoreductases, and the biosynthesis of 1 mol of (*S*)-equol necessitates the consumption of 2 mol of NADPH [8,12]. Therefore, the conversion of daidzein to (*S*)-equol will be impaired when intracellular NADPH is insufficient [8].

Quorum sensing (QS) is vital in bacterial physiological processes, allowing bacteria to adapt to environmental changes and explore new environmental niches [13]. This communication significantly impacts exogenous probiotic metabolism and phenotype, influencing traits such as acid resistance, adhesion, and intestinal colonization [14,15]. The LuxR/I-type QS systems are commonly seen in most Gram-negative proteobacteria, where the QS signal is provided by acyl homoserine lactone (AHL) [13]. Typically, these bacteria encode LuxI synthase, for AHL production, and the cognate LuxR transcription factor, which is regulated by AHL [13,16]. However, in EcN, only the LuxR-type transcription factor SdiA is present without the LuxI-type synthase, which functions in intraspecies and interspecies communication by detecting exogenous AHLs from other bacteria [16,17,18]. Recent studies found that AHL-QS could regulate NADPH metabolism in microorganisms by regulating the glucose-6-phosphate dehydrogenase and pentose phosphate pathways [19,20,21,22].

The impact of intestinal flora on engineered EcN and the biosynthesis of (*S*)-equol, particularly in relation to QS regulation, remains largely unexplored, despite the documented production of AHLs by intestinal flora [16]. Therefore, the aims of this study were (1) to investigate the influence of gut microbiota on NADPH metabolism, with a specific focus on the role of the QS transcription factor SdiA, and (2) to elucidate the molecular mechanism by which AHL-SdiA regulates NADPH metabolism, with the aim of optimizing the (*S*)-equol production of engineered EcN strains in the intestinal environment. These results could clarify the effect of AHL-QS of gut microbiota on the NADPH metabolism of EcN, which is instructive for developing engineered probiotics suited to the gut environment.

## 2. Materials and Methods

### 2.1. Strains and Chemicals

*E. coli* DH5α was utilized for plasmid amplification, while EcN was employed for gene expression. A comprehensive list of all strains used in this investigation is presented in Table 1. All genetic manipulations, including the use of restriction enzymes, DNA ligases, High-Fidelity DNA polymerase, and one-step directional cloning kits, were sourced from Yeasen Biotech (Shanghai, China). Standard and commercial soy isoflavones (SI; 80% *w*/*w* isoflavones) were purchased from Yuanye Biotech (Shanghai, China). The isoflavones in the SI used in this study comprised 522.35 μg/mg of daidzin, 184.72 μg/mg of glycitin, 77.51 μg/mg of genistin, 20.38 μg/mg of daidzein, 10.05 μg/mg of glycitein, and 6.17 μg/mg of genistein.

### 2.2. Plasmids and DNA Manipulation

The specifics of the primers and plasmids utilized in this study are outlined in Tables S1 and S2. The synthesis of *dznr* from *Asaccharobacter celatus*, as well as *ddrc*, *thdr*, and *dhdr* from *Slackia isoflavoniconvertens*, was performed by Sangon (Shanghai, China), incorporating codon optimization for *E. coli* [3,8]. For the construction of pETM6-*Pnar*, the *nar* promoter sequence (*Pnar*) was chemically synthesized, aligning with previously reported sequences [3]. The *Avr*II- and *Nde*I-digested *Pnar* fragment was subsequently cloned into the *Avr*II/*Nde*I-digested pETM6 plasmid, creating pETM6-*Pnar*. To obtain the *Pnar* sequence, the primers Pf_Pnar and Pr_Pnar were employed in a PCR reaction. The resulting PCR product mixture underwent *Dpn*I digestion and subsequent ligation using one-step cloning kits.

The *malEK* and *exo/cea* locus of EcN were selected as suitable sites for transgene integration and expression [23,24]. Subsequently, EcN genomic DNA served as a template for amplifying the corresponding homology arm fragments via PCR. *Avr*II and *Sal*I restriction sites were introduced at the upstream homology arm to facilitate further manipulations during the PCR amplification. These resulting DNA fragments were efficiently cloned into the pUC57 vector using one-step cloning kits, creating pUC57-*malEK* and pUC57-*exo/cea*.

Primer pairs consisting of Pf_bglF/Pr_bglF and Pf_bglB/Pr_bglB were employed to clone *bglF* and *bglB* from *E. coli* K12 genomic DNA into pETM6-*Pnar*, resulting in the construction of pETM6-*Pnar*-*bglF-Pnar-bglB*. The plasmid pUC57-*exo/cea-Pnar-bglF-Pnar-bglB*, harboring donor DNA fragments, was established by integrating the appropriate fragments into the *Avr*II/*Sal*I-digested pUC57-*exo/cea*. The donor DNA was obtained through PCR amplification. To mediate the integration of the homologous arm into the chosen locus, transformation was carried out using the Cas9-recombinase-expressing plasmids pEcCas and pTarget [25,26]. Specific sgRNAs for the *malEK* and *exo/cea* loci were identified and ranked within the EcN genetic background using online software (https://chopchop.cbu.uib.no/# (accessed on 19 November 2023)) [27]. For the creation of the EcN-eq strain, *Nde*I/*Kpn*I-digested synthesized DNA fragments were cloned into the *Nde*I/*Kpn*I-digested pETM6-*Pnar*. Intermediate plasmids resulting from this process were subsequently digested with *Avr*II and *Sal*I, and the resulting DNA fragments were inserted into *Spe*I/*Sal*I sites of the corresponding plasmids using BioBrick cloning [28]. This led to the development of pETM6-*Pnar-dznr-Pnar-ddrc-Pnar-dhdr-Pnar-thdr.* The quadruple-gene cassette was integrated into pUC57-*exo/cea*, and the donor DNA was incorporated into the *exo*/*cea* locus following the previously described method. CRISPR/Cas9-mediated gene deletions were employed to implement the relevant gene knockouts, with a constitutively expressed kanamycin resistance gene introduced during the homologous recombination of the inactivated *ptsG*.

In order to facilitate the overexpression of *decR*, *HW372_01960*, *yhjC*, *HW372_03545*, *sdiA*, and *yhaJ*, each gene was individually amplified from the EcN genome using their respective primers. Subsequently, each gene was introduced into pETM6-*Pnar* using one-step cloning kits.

### 2.3. Culture Conditions

Luria–Bertani (LB) medium served as the cultivation medium for *E. coli* cells during gene cloning, plasmid propagation, and inoculum preparation. A single colony was selected and cultured in LB medium, followed by overnight incubation at 37 °C with shaking at 220 rpm. For in vitro fermentation experiments involving engineered EcN strains, a basal nutrient medium was utilized. The stock basal nutrient medium, with a pH of 7.0, consisted of peptone (10.00 g/L), yeast extract (10.00 g/L), NaHCO_3_ (10.00 g/L), glucose (5.00 g/L), bile salts (2.50 g/L), L-cysteine hydrochloride (2.50 g/L), NaCl (0.150 g/L), K_2_HPO_4_ (0.2 g/L), KH_2_PO_4_ (0.2 g/L), heme (0.2 g/L), MgSO_4_ (0.05 g/L), CaCl_2_ (0.05 g/L), resazurin (5 mg/L), Tween 80 (10 mL), and vitamin K1 (50 μL) [29]. Before use, the stock medium was diluted at a ratio of 1:5.

Human fecal samples were voluntarily provided by eight healthy young donors (four females and four males) who refrained from antibiotic use for at least four months and who had no history of gastrointestinal disorders. Each fresh fecal sample was collected in stool tubes and combined with a 1:2 (*w*/*v*) ratio of modified sterile saline (9.0 g/L NaCl and 0.5 g/L cysteine-HCl) under anaerobic conditions in an anaerobic glove box (Xinmiao YQX-11, Shanghai, China). Fecal supernatants were obtained by centrifugation at 300 rpm for 20 min, followed by thorough mixing in equal proportions. Sterile fecal filtrates were then acquired by filtering the fecal supernatant through a sterile 0.2 μm membrane. Subsequently, the stock basal nutrient medium was blended with either fecal supernatants or sterile fecal filtrates at a volume ratio of 1:4. The initial inoculum of engineered EcN was set at 2 × 10^7^ CFU/mL and incubated at 37 °C within an anaerobic glove box. To extract reaction samples, two volumes of ethyl acetate were used, and isoflavonoids were concentrated using a rotary evaporator for subsequent chromatographic separation.

### 2.4. SDS-PAGE Gel Electrophoresis

Following incubation, the cultured cells were retrieved through centrifugation at 9500× *g* for 10 min at 4 °C and subsequently resuspended in 1 mL of PBS buffer. In order to prepare the samples for electrophoresis, 0.5 mL of the suspension was combined with 0.5 mL of 2× SDS-PAGE sample loading buffer (Beyotime, Shanghai, China), boiled for 10 min, allowed to cool, and then subjected to centrifugation at 11,500× *g* for 2 min. Then, 20 μL of the resulting samples were applied to pre-cast 12% gels from Yeasen (Shanghai, China). Proteins were separated by electrophoresis at 120 V for a duration of 60–90 min.

### 2.5. Determination of Intracellular NADPH Levels

The conversion of OD_600_ values to cell numbers was achieved through correlation with colony counting, and the cell densities were subsequently obtained by reading the OD_600_. A total of 4–5 million cells were collected through centrifugation (2 min, 13,000× *g*). Following the manufacturer’s instructions, the cell precipitates were washed using a mixed solution (70 mM HEPES, 60% MeOH) and resuspended in 0.9 mL of the acid extraction buffer (Beyotime, Shanghai, China) from the kit. The cell suspension underwent sonication for 1 min and was then centrifuged at 10,000× *g* for 10 min at 4 °C. The supernatant (200 μL) was transferred to a new centrifuge tube, and an equal volume of alkaline buffer (Beyotime, Shanghai, China) was added to neutralize it. This mixture was then centrifuged at 12,000× *g* at 4 °C for 10 min, and the resulting supernatant was collected. The quantification of NADP^+^ and NADPH was performed using the NADP^+^/NADPH Assay Kit from Beyotime, China.

### 2.6. Transcriptome Analysis by RNA-seq

The cultures underwent a 2 h incubation at 37 °C prior to RNA extraction. For this, 10 mL of collected cells were rapidly frozen in liquid nitrogen, and total bacterial RNA was extracted using the RNAprep pure Kit (TIANGEN, Beijing, China). RNA quality was checked by 1% agarose gel electrophoresis to verify the integrity of the RNA preparations. The extracted RNA was then transported on dry ice to Sangon Biotech (Shanghai, China) for transcriptome re-sequencing analysis. To obtain mRNA, the total RNA underwent rRNA removal, and the resultant mRNA was utilized as a template for DNA synthesis. Sequencing of the cDNA libraries was performed using the Illumina HiSeq platform. For annotation purposes, the EcN genome served as a reference. Differentially expressed genes between the sample and engineered strains were determined based on a false discovery rate (FDR)  ≤  0.05 and a fold change (log2Ratio) ≥ 2. To categorize genes at the KEGG_B_class level, the pathway-enrichment analysis tool Omicshare was employed.

### 2.7. Real-Time PCR Measurements for Transcriptional Analysis

Real-time PCR was employed to assess the transcriptional expression levels of the targeted genes. The initial isolation of total RNA from recombinant cells was performed using the MolPure Bacterial RNA Kit from Yeasen (Shanghai, China). The subsequent synthesis of cDNA was achieved using the Strand cDNA Synthesis SuperMix for qPCR, also from Yeasen. For the real-time PCR analysis, the LightCycler 480 Real-Time PCR System from Roche (Mannheim, Germany) was utilized, along with the qPCR SYBR Green Master Mix. Quantitative PCR amplification was conducted using 2 μL of diluted cDNA template. The final concentration of primers in the reaction solution was 0.2 μM, and the total volume was adjusted to 20 μL with ddH_2_O. The amplification conditions included an initial predenaturation step at 95 °C for 2 min, followed by a two-step reaction (95 °C, 10 s; 60 °C, 30 s) for 40 cycles. The 16S gene was chosen as the endogenous reference gene to determine the relative mRNA expression levels of *zwf* (glucose-6-phosphate dehydrogenase) and *gnd* (6-phosphogluconate dehydrogenase), applying the 2^−ΔΔCt^ method [25].

### 2.8. Molecular Docking

The prediction of the SDH structure was conducted using AlphaFold2 through Google Colab [30]. Molecular docking procedures involving SdiAs with C10-HSL were carried out using the Discover Studio 2021 Libdock module. The docking pose selected adhered to the distance restraints of SdiAs with C10-HSL, emphasizing the highest docking score and the absence of adverse contacts. Analysis of the docking-complex structure was conducted utilizing the open-source PyMol.

### 2.9. MD Simulations

The MD simulations were carried out using GROMACS 2022 to analyze heat fluctuations. In brief, the protein structure was treated with the Charmm36 force field. Each system’s proteins were solvated in the TIP3P water box. The MD simulations were conducted at 310 K for 100 ns. Energy minimization involved the use of the steepest descent for 1000 steps, followed by gradual heating of each system to 310 K and equilibration for 100 ps NVT and 100 ps NPT. The temperature was set to 310 K with a time constant of 0.1 ps, utilizing a V-rescale Berendsen thermostat. The pressure was maintained at 1 bar with a time constant of 2 ps, employing Parrinello–Rahman pressure coupling. Subsequently, the simulations were conducted for 100 ns, with results saved at intervals of 2 fs. The analysis of simulated proteins involved studying the root-mean-square deviation (RMSD), root-mean-square fluctuation (RMSF), and hydrogen-bond numbers using gmx-rms, gmx-rmsf, and gmx-gyrate, respectively.

### 2.10. Analytical Methods

Isoflavonoids were analyzed using the Agilent 1260 series HPLC system equipped with an Agilent ZORBAX SB-C18 column (4.6 × 250 mm, 5 μm). The separation of isoflavonoids was conducted with solvent A (containing 0.1% formic acid) and solvent B (methanol). The gradient elution program and detection methodology followed a previously established method [5]. For chiral analysis, the CHIRALPAK-IC column from Daicel Chemical Industries (4.6 mm × 150 mm, 5 μm) was utilized with an isocratic mobile phase of 1 mL/min (hexane/ethanol = 70:30) [3].

Reversed-phase solid-phase extraction was employed for the extraction of AHLs from feces [16]. In this process, each 100 µL sample was combined with 100 µL of a 100 nM C6-HSL solution (in methanol) and 1600 µL of cold methanol (0.1% formic acid). Following vortex mixing for 15 s, the tubes were placed at −20 °C for 30 min and then centrifuged at 18,000× *g* for 10 min. The supernatants were collected and subsequently speed-vacuum-dried. Subsequently, 200 µL of methanol was added to the tube and vortex mixed for 30 s, followed by the addition of 1.5 mL of water (0.1% formic acid). The resulting mixture was loaded onto an ISOLUTE C18 cartridge (100 mg/1 mL). After vacuum drying, the analytes were eluted with 1500 µL of acidified methanol (0.1% formic acid). The eluates were speed-vacuum-dried at 8 °C and then resuspended in 200 µL of 50% methanol (0.1% formic acid). Analysis was performed using a UHPLC system (Agilent, Santa Clara, CA, USA) coupled with a Quantis triple quadrupole mass spectrometer via an electrospray ionization (ESI) source operated in the positive ion mode. The T3 (2.1 mm × 100 mm, 1.8 µm) column from Waters (Milford, MA, USA) was used for chromatographic separation, with mobile phase A consisting of 0.1% formic acid in water and mobile phase B consisting of 0.1% formic acid in acetonitrile. The AHLs were eluted at a flow rate of 200 µL/min using a gradient elution of the mobile phase starting at 5% B and then increasing to 10% B by 1 min, further increasing to 75% B by 3 min, and reaching 99% B by 6 min, which was held until 6.5 min; this was then decreased to 5% B by 13 min and held for 3.5 min, resulting in a total run time of 16.5 min. The injection volume was 10 µL, and the column temperature was maintained at 50 °C. Source parameters for the mass spectrometer were set as follows: spray voltage, 4500 V; ion-transfer-tube temperature, 350 °C; vaporizer temperature, 450 °C.

### 2.11. Statistical Analysis

We conducted statistical analyses using SPSS v26.0 (SPSS, Inc., Chicago, IL, USA). To assess statistical significance, we employed the Student’s *t*-test, denoting significance levels as follows: * *p* < 0.05, ** *p* < 0.01, and *** *p* < 0.001. The error bars in the figures represent the standard deviation (SD).

## 3. Results and Discussion

### 3.1. Engineering E. coli Nissle 1917 for (S)-Equol Production from Daidzein and Daidzin through Chromosomal Integration

**Figure 1:**
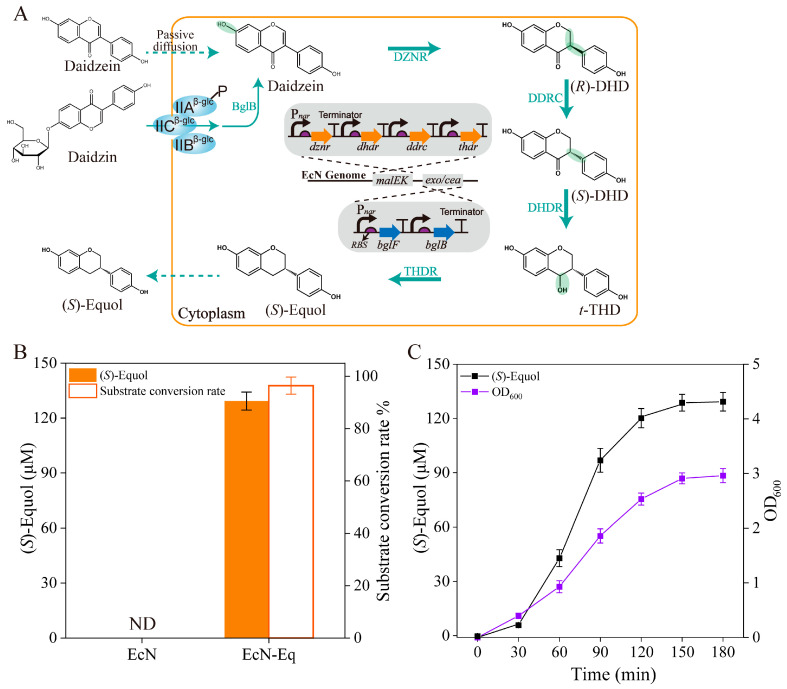
Construction of the EcN-eq strain for the (*S*)-equol conversion from daidzin and daidzein. (**A**) Illustration outlining the gene constructs incorporated into the EcN-eq strain, developed by gene insertion into the EcN genome. The green color represents the moiety of the structural formula that has changed. (**B**) Utilization of whole-cell cultures of the EcN-eq strain for the conversion of daidzin and daidzein into (*S*)-equol. A substrate of 100 mg/L of SI was introduced into the medium. (**C**) Representation of the growth curve of the EcN-eq strain and its temporal profile for (*S*)-equol production. All experiments were performed in triplicate, and error bars indicate the standard deviation (SD) with a 95% confidence interval (CI).

Isoflavones in soy products are in the form of glycoside isoflavones (e.g., daidzin), which need to be hydrolyzed to isoflavone aglycones (e.g., daidzein) to be utilized by *E. coli* [2,3,31,32]. Building upon a prior study where recombinant *E. coli* strains expressing both the β-glucoside influx transporter (BglF) and 6-phospho-β-glucosidase (BglB) from *E. coli K12* showcased the ability to utilize daidzin, we opted for the introduction of *bglF* and *bglB* to enable daidzin utilization in EcN [3]. Subsequently, *dznr* from *Asaccharobacter celatus*, along with *ddrc*, *thdr*, and *dhdr* from *Slackia isoflavoniconvertens* were selected to convert daidzein into (*S*)-equol based on their efficient (*S*)-equol-production performance [8]. To regulate exogenous gene expression within the anaerobic gut environment, the anaerobically induced promoter *Pnar* was employed due to its functional adaptability across various growth phases of engineered *E. coli* strains [3,33]. These six genes underwent chromosomal integration and were transcriptionally regulated by the *Pnar* promoter, thereby generating the strain EcN-eq (Figure 1A). The (*S*)-equol-producing capacity of EcN-eq was examined in basal nutrient medium containing 100 mg/L soy isoflavone (SI; 80% *w*/*w* isoflavone), resulting in the generation of 129.3 μM of (*S*)-equol with a conversion rate of 96.4% (mol/mol) after 150 min of bioconversion (Figure 1B,C). The chiral HPLC analysis determined the stereochemical configuration of (*S*)-equol, with the enantiomeric excess values exceeding 99.0% (*S*) (Figure S1).

### 3.2. (S)-Equol Production by the EcN-eq Strain in Media with Fecal Supernatants or Sterile Fecal Filtrates

**Figure 2:**
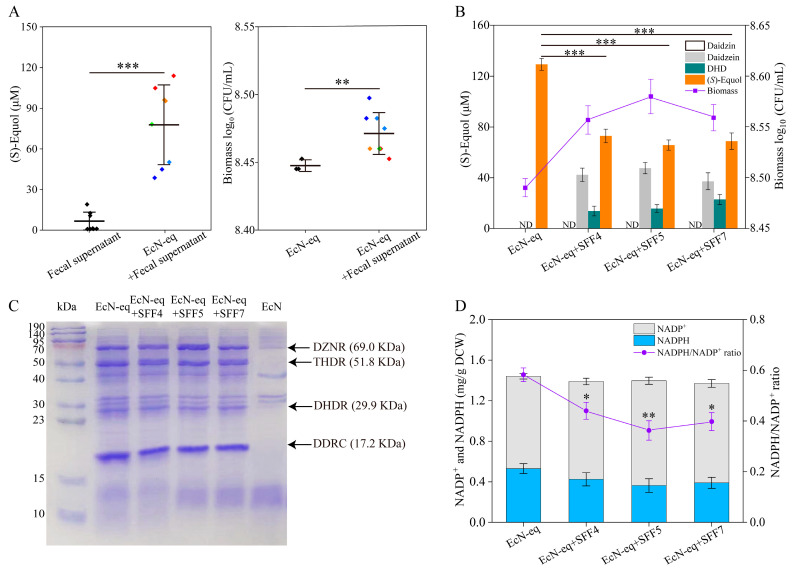
(*S*)-Equol production by the EcN-eq strain in media with fecal supernatants or sterile fecal filtrates. (**A**) Biomass and (*S*)-equol titer of the EcN-eq strain in media containing fecal supernatants. (**B**) Yield of (*S*)-equol and intermediates of the EcN-eq strain in media containing SFF4, SFF5, and SFF7, respectively. (**C**) Expression analysis of enzymes involved in the (*S*)-equol biosynthetic pathway by the EcN-eq strain in media containing SFF4, SFF5, and SFF7, respectively. (**D**) NADPH and NADP^+^ content of the EcN-eq strain in media containing SFF4, SFF5, and SFF7, respectively. EcN-eq served as the control. Statistical analysis was conducted using a *t*-test, where * *p* < 0.05, ** *p* < 0.01, and *** *p* < 0.001. Error bars indicate the standard deviation (SD).

The efficient EcN-eq strain, developed for (*S*)-equol production from SI via the daidzin-utilization pathway and the (*S*)-equol biosynthesis pathway in EcN, was initially tested under optimal growth conditions in monoculture. However, the outcomes might not accurately represent real-life scenarios [34]. To assess the potential of EcN-eq as a cooperative blend of probiotics in conferring metabolic pathways for converting dietary SI into (*S*)-equol, we examined its (*S*)-equol production in a basal nutrient medium containing human fecal supernatant in vitro. Human fecal samples were voluntarily provided by eight healthy young donors (four females and four males) who had refrained from antibiotic use for at least four months and who had no history of gastrointestinal disorders. As depicted in Figure 2A, when EcN-eq was absent in the fecal supernatant cultures, (*S*)-equol was detected in only three samples, reaching a maximum titer of 18.7 μM. In contrast, the presence of EcN-eq in the fecal-supernatant cultures led to a significantly higher (*S*)-equol titer, measuring 77.5 μM compared with the control. However, the (*S*)-equol production by EcN-eq when co-cultured with gut microorganisms was 40.1% lower than in monoculture, displaying substantial variability among individuals (Figure 1C and Figure 2A). Intriguingly, the biomass of EcN-eq co-cultured with gut microorganisms was significantly higher than that of EcN-eq in monoculture (Figure 2A).

The interaction between gut microbiota and probiotics often occurs through microbial metabolites, exerting an influence on their metabolism and phenotype [14,35]. To identify the bottleneck in EcN-eq’s performance when co-cultured with fecal bacteria, we scrutinized the intermediates and (*S*)-equol production during the whole-cell reaction of EcN-eq in cultures containing sterile fecal filtrates (i.e., SFF4, SFF5, and SFF7). Consistent with findings from the fecal-supernatant cultures, the presence of SFF4, SFF5, and SFF7 led to a notable reduction in (*S*)-equol production by EcN-eq compared with cultures without SFFs (Figure 2B). Remarkably, in these cultures, the major intermediates observed were daidzein and dihydrodaidzein (DHD), while daidzin was notably absent. Moreover, EcN-eq displayed a substantial increase in biomass in media containing SFF4, SFF5, and SFF7 relative to cultures without SFFs (Figure 2B). From these observations, we hypothesized that metabolites present in fecal-filtrate cultures potentially diminish the (*S*)-equol production titer of EcN-eq by impeding the conversion of daidzein into (*S*)-equol within EcN-eq.

Based on the above inference, we proceeded to examine the expression of pathway enzymes responsible for converting daidzein into (*S*)-equol in EcN-eq within the SSF-added media. As shown in Figure 2C and Figure S2, the supplementation of SSF in the medium did not significantly impact the expression of DZNR, DDRC, DHDR, and THDR in EcN-eq. Given that DZNR and DHDR rely on NADPH as a coenzyme for their enzymatic activity [36,37], we further investigated the effects of SSF addition to the media on EcN-eq’s intracellular NADPH levels and the NADPH/NADP^+^ ratio (Figure 2D). The results revealed a significant decrease in EcN-eq’s intracellular NADPH levels and NADPH/NADP^+^ ratio upon the addition of SSF to the media. Notably, the declining trend in the NADPH/NADP^+^ ratio corresponded consistently with the reduction observed in the (*S*)-equol titer (Figure 2B,D). NADPH is a pivotal component for biosynthesizing cellular components within the cell, while also serving as a crucial cofactor in producing various nutraceuticals and fine chemicals [38,39]. Notably, previous research has illustrated that engineered *E. coli* strains possess the capability to effectively convert daidzein (ranging between 197–500 μM) into (*S*)-equol using endogenous NADPH [3,40]. While enhancing intracellular NADPH levels can be achieved through the overexpression of enzymes responsible for its regeneration, this approach may disrupt the balance between oxidized and reduced forms of the cofactors [41]. Consequently, optimizing the endogenous NADPH regeneration pathway within the strain holds promise for enhancing the efficient synthesis of desired products.

### 3.3. Transcriptome Analysis of the EcN-eq Strain in Media with or without the Addition of Sterile Fecal Filtrates

**Figure 3:**
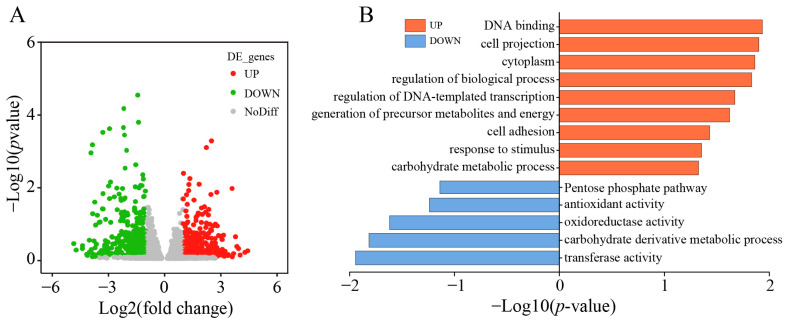
Transcriptome analysis of the EcN-eq strain in media with or without the addition of SFF5. (**A**) Volcano plot of differentially expressed genes in the EcN-eq strain in medium with or without added SFF5. (**B**) KEGG pathway-enrichment map, where the *X*-axis is the negative logarithmic transformation of *p*-values and the *Y*-axis is the enriched pathway; blue bars indicate pathways enriched by downregulated proteins, and red bars indicate pathways enriched by upregulated proteins.

To further elucidate the influence of metabolites present in fecal-filtrate cultures on the regulation of intracellular NADPH metabolism in EcN-eq, we conducted RNA-Seq analyses on EcN-eq samples that had been fermented for 60 min in media both with and without SSF5 addition. Genes exhibiting an absolute log2-fold change greater than 2 (with a *p*-value < 0.05) were categorized as differentially expressed genes (DEGs). Transcriptomic data indicated that 29 genes were upregulated while 17 genes were downregulated when comparing EcN-eq cultured in SSF5-containing medium to those in medium lacking SSF5 (Figure 3A). The identified DEGs were categorized by KEGG analysis (Figure 3B). The DEGs were predominantly associated with biological processes such as oxidoreductase activity, carbohydrate-derivative metabolism, and NADPH metabolism. Notably, genes involved in DNA binding, cell projection, and regulation of DNA-templated transcription showed increased expression, while those linked to the pentose phosphate pathway, oxidoreductase activity, transferase activity, and carbohydrate-derivative metabolic processes exhibited decreased expression (Figure 3B). As can be seen from Table 2, there were significant variations in the expression levels of six transcription factors (TFs) in EcN-eq when cultured in SSF5-containing medium. These TFs have the potential to modulate intracellular NADPH levels directly or indirectly.

### 3.4. Quorum-Sensing Transcription Factor SdiA Modulates the Intracellular NADPH/NADP^+^ Ratio

**Figure 4:**
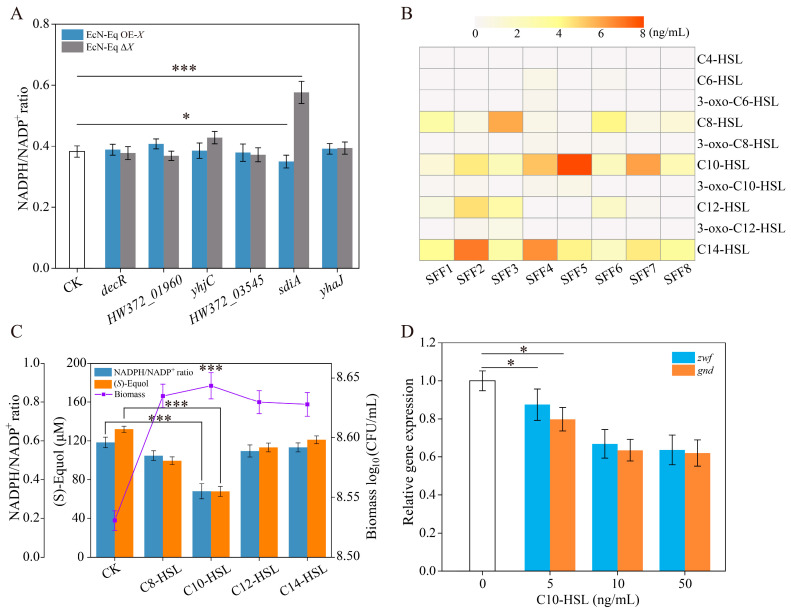
The SdiA quorum-sensing transcriptional regulator modulates the intracellular NADPH/NADP^+^ ratio in the EcN-eq strain. (**A**) Determination of NADPH/NADP^+^ ratios in transcription-factor-overexpression strains (EcN-eq OE-*X*) and knockout strains (EcN-eq Δ*X*). (**B**) Determination of N-acyl homoserine in SFF1 to SSF8 samples. (**C**) Effect of medium- and long-chain N-acyl homoserine lactones (C8-HSL to C14-HSL) on the NADPH/NADP^+^ ratio, (*S*)-equol titer, and biomass in the EcN-eq strain. The concentration of N-acyl homoserine lactones was 8 ng/mL. (**D**) Effect of different concentrations of C8-HSL on *zwf* and *gnd* gene expression in the EcN-eq strain. Statistical analysis was performed using a *t*-test; * *p* < 0.05, *** *p* < 0.001, and the error bars represent the standard deviation (SD).

In order to elucidate the functional roles of the aforementioned transcriptional regulators, additional validation was conducted. This involved the construction of EcN-eq OE-*X* strains, characterized by the overexpression of the TFs, and EcN-eq Δ*X* strains, in which the TFs were knocked out. Subsequently, the intracellular NADPH/NADP^+^ ratios were measured in media containing SSF5. As shown in Figure 4A, the intracellular NADPH/NADP^+^ ratio of the EcN-eq strain Δ*sdiA* was significantly higher compared with the control, whereas the intracellular NADPH/NADP^+^ ratio of the EcN-eq strain OE-*sdiA* was lower than that of the control. SdiA is a LuxR-type transcription factor that is recognized as a sensor for *E. coli* to detect AHLs produced by other bacteria in the environment [13]. These results strongly suggest the involvement of the QS transcription factor SdiA in regulating NADPH metabolism within EcN-eq.

Since SdiA recognizes a broad spectrum of AHLs, varying in acyl chain length and oxidation [17,18], we employed liquid mass spectrometry to quantify the species and concentrations of AHLs present in the SFFs. Table S2 exhibits the MRM transitions of the AHLs measured in this study. Figure 4B illustrates the substantial diversity in AHL types and concentrations across the eight SFF samples. Notably, AHLs with short side-chains (C_4–6_-HSL) were absent in all samples, while AHLs featuring long side-chains (C_8–14_-HSL) were detectable in each sample (Figure 4B). A previous study indicated distinct differences in SdiA complex formation involving different AHLs, resulting in the differential regulation of target genes within the *E. coli* genome [18]. In light of this, we proceeded to explore the impact of C8-HSL, C10-HSL, C12-HSL, and C14-HSL on EcN-eq’s intracellular NADPH/NADP^+^ ratio and biomass. Notably, Figure 4C reveals that only the addition of C10-HSL in the medium elicited a significant reduction in EcN-eq’s intracellular NADPH/NADP^+^ ratio and (*S*)-equol titer among the evaluated AHL candidates. Moreover, these AHLs contributed to a notable increase in the EcN-eq biomass compared with the control. However, no substantial differences were observed in EcN cultures supplemented with various AHLs.

In *E. coli* strains, NADPH production primarily occurs through the oxidative branch of the pentose phosphate pathway, wherein glucose-6-phosphate dehydrogenase (encoded by *zwf*) and 6-phosphogluconate dehydrogenase (encoded by *gnd*) play crucial roles in NADPH regeneration [8]. Transcriptomic analysis revealed a downregulation of both *zwf* and *gnd* expression in EcN-eq when supplemented with SSF5 (Table 2). To elucidate the regulatory role of the C10-HSL-SdiA complex on *zwf* and *gnd*, we assessed the impact of varying concentrations of C10-HSL (ranging from 0 to 50 ng/mL) in the culture medium on the expression of *zwf* and *gnd* in EcN-eq using RT-qPCR (Figure 4D). The results indicated that even at a low C10-HSL concentration of 5 ng/mL, there was a negative modulation of *zwf* and *gnd* expression in EcN-eq. Furthermore, higher concentrations of C10-HSL (10 and 50 ng/mL) led to a further decline in *zwf* and *gnd* expression in EcN-eq, reaching approximately 35% lower expression levels compared with the control (Figure 4D). In summary, the QS transcription factor SdiA appears to exert a negative regulatory effect on *zwf* and *gnd* in EcN-eq, potentially resulting in decreased intracellular NADPH levels.

### 3.5. Interaction Analysis of N-Decanoyl-l-Homoserine Lactone (C10-HSL) with SdiA_EcN

*E. coli* regulates growth and metabolic processes through SdiA in response to AHLs produced by other bacteria, enabling *E. coli* to navigate environmental variations and explore new ecological niches [18,42,43]. However, limited information exists on the role of SdiA in regulating intracellular NADPH metabolism in EcN. A previous study has shown that the N-terminal ligand-binding structural domain (LBD) of SdiA from the model organism *E. coli K12* has constrained accommodation for AHLs with acyl chain lengths beyond C8 [42]. Consequently, variations in the affinities of SdiA from different sources for long-side-chain AHLs could result in differential metabolic regulation by SdiA [18]. Given that EcN serves as a representative chassis for engineered probiotics, elucidating the disparities in C10-HSL interactions with SdiA_EcN and SdiA_K12 from *E. coli K12* could aid in developing engineered EcN strains tailored to the intestinal environment.

**Figure 5:**
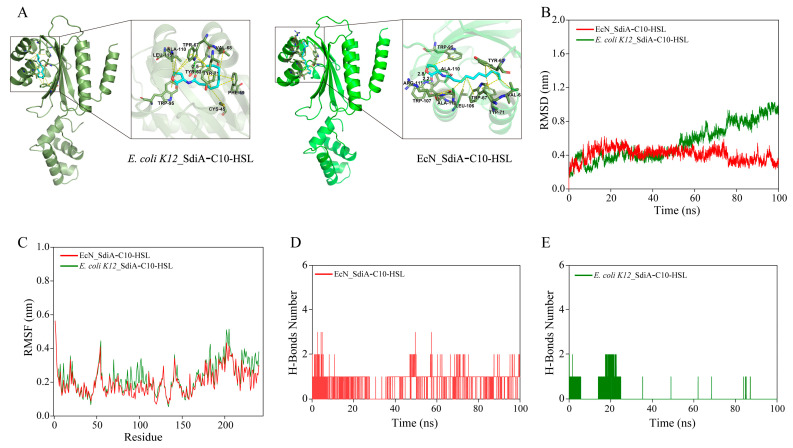
Molecular-docking analysis and MD simulation analysis of *E. coli K12*_SdiA and EcN_SdiA with C10-HSL. (**A**) Molecular-docking analysis of *E. coli K12*_SdiA and EcN_SdiA with C10-HSL. (**B**) RMSD analysis of *E. coli K12*_SdiA and EcN_SdiA with C10-HSL. (**C**) RMSF analysis of *E. coli K12*_SdiA and EcN_SdiA with C10-HSL. (**D**) Time profiles of hydrogen bonding of *E. coli K12*_SdiA with C10-HSL. (**E**) Time profiles of hydrogen bonding of EcN_SdiA with C10-HSL.

Given the unavailability of the crystal structure for SdiA_EcN, we employed AlphaFold2 for predictive modeling [30]. SdiA_EcN and SdiA_K12 exhibit structural resemblances, encompassing an AHL-binding ligand-binding domain (LBD) (Figure 5A). This domain adopts an alpha–beta–alpha sandwich configuration. Additionally, both share a C-terminal domain that functions as a DNA-binding structural domain, comprising four helices and featuring a helix–turn–helix motif for DNA binding (Figure 5A). Molecular-docking analysis revealed insights into the interactions between SdiA and C10-HSL at a molecular level (Figure 5A). The conformation of C10-AHL-SdiA_EcN is similar to that of C10-AHL-SdiA_K12, showcasing the stabilization of the acyl chain via hydrophobic residues [42]. However, notable distinctions arise in the hydrogen-bond interactions involving the lactone ring of C10-AHL and residues in SdiA_EcN versus SdiA_K12. In the docked conformation of SdiA_EcN with C10-AHL, evident hydrogen bonds form between W107 and the lactone ring, as well as between R111 and the lactone ring’s carbonyl group. Conversely, in SdiA_K12, the hydrogen bond solely occurs between Y63 and the C1 carbonyl group. Literature reports indicate that mutations in specific distal or surface amino acid residues can alter the electrostatic charge distribution on the surface, affecting the LBD’s interaction with ligands [44,45]. Therefore, the mutation of hydrophobic amino acid residue A66, situated in the alpha helix on the surface of SdiA_K12, to the hydrophilic amino acid S66 in SdiA_EcN may potentially induce variations in LBD volume, thereby influencing the differential binding of SdiA_EcN and SdiA_K12 to C10-HSL (Figure S3).

The molecular-docking findings were validated through an additional 100 ns of molecular-dynamics simulations. The root-mean-square deviation (RMSD), a pivotal parameter for assessing conformational fluctuations within the protein–ligand complex, inversely correlates with complex stability [46]. Notably, during the 60–100 ns period, the RMSD values of C10-HSL-SdiA_EcN exhibited lower values compared with C10-HSL-SdiA_K12, implying heightened thermal stability within the C10-HSL-SdiA_EcN complex (Figure 5B). Furthermore, examining the root-mean-square fluctuation (RMSF) values of residues 75–110 within the LBD revealed lower fluctuations in the C10-HSL-SdiA_EcN complex compared with C10-HSL-SdiA_K12, indicating reduced flexibility in this region for the former (Figure 5C). Quantification of hydrogen bonding at the binding site was performed to elucidate the nature of interactions between C10-HSL and SdiAs. Throughout the simulation, the number of stable hydrogen bonds between SdiA_EcN and C10-HSL remained consistently around one, with a maximum of three hydrogen bonds formed, indicative of a more stable binding between SdiA_EcN and C10-HSL (Figure 5D). Conversely, minimal stable hydrogen bonds were observed between SdiA_K12 and C10-HSL, suggesting the lack of a stable conformation within the C10-HSL-SdiA_K12 complex (Figure 5E).

### 3.6. Replacing sdiA_EcN with sdiA_K12 Improved (S)-Equol Production and Avoided Biomass Reduction

**Figure 6:**
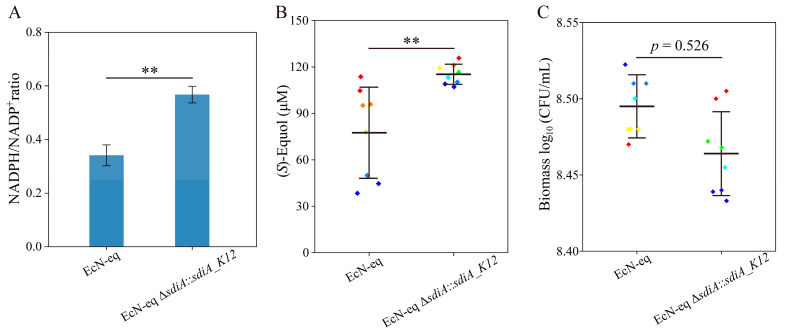
(**A**) NADPH/NADP^+^ ratio of the EcN-eq and EcN-eq *sdiA*::*sdiA_K12* strains in media containing fecal filtrates. (**B**,**C**) (*S*)-Equol production titers (**B**) and biomass (**C**) of the EcN-eq strain *sdiA*::*sdiA_K12* in media containing fecal supernatants. Statistical analysis was performed using a *t*-test; ** *p* < 0.01, and the error bars represent the standard deviation (SD).

Given the global gene regulation and growth-promoting role of *sdiA* in EcN, we replaced *sdiA_EcN* with *sdiA_K12* to elevate the intracellular NADPH levels within EcN-eq, aiming to enhance its (*S*)-equol biosynthesis. The construction of the EcN-eq strain Δ*sdiA*::*sdiA_K12* involved the replacement of *sdiA_EcN* coding sequences with *sdiA_K12* utilizing CRISPR/Cas9- and λ-Red-mediated recombination [3,26]. Subsequently, the intracellular NADP^+^/NADPH ratios were assessed for both EcN-eq Δ*sdiA*::*sdiA_K12* and EcN-eq in media containing fecal filtrates. As shown in Figure 6A, the intracellular NADPH/NADP^+^ ratio of EcN-eq Δ*sdiA*::*sdiA_K12* exhibited a significant increase compared with that of EcN-eq. Following a 150 min biotransformation period, the (*S*)-equol titer of EcN-eq Δ*sdiA*::*sdiA_K12* reached 113.7 μM with an 84.8% substrate conversion rate, indicating a 47% increase compared with EcN-eq (Figure 6B). Furthermore, the EcN-eq Δ*sdiA*::*sdiA_K12* group displayed a lower standard deviation in the (*S*)-equol titer than the EcN-eq group and showed no significant difference in biomass (Figure 6B,C).

Isoflavones are commonly utilized as antioxidative dietary supplements. However, (*S*)-equol, a secondary metabolite of isoflavone by the gut microbiota, exhibits significantly higher antioxidative capacity than its precursor [2,4]. Consequently, developing EcN strains with the ability to efficiently convert dietary isoflavones into (*S*)-equol may represent an effective strategy to confer the (*S*)-equol-producing phenotype of the host. Nevertheless, the intricacies of the gut microbiome may have contributed to the challenges encountered in in vivo studies involving engineered EcN strains [47]. This study offers insights into the development of (*S*)-equol-producing EcN probiotics tailored for the intestinal environment by elucidating the impact of AHL-SdiA on EcN NADPH metabolism.

## 4. Conclusions

This study delves into how AHL-SdiA-mediated QS diminishes (*S*)-equol production by modulating NADPH metabolism in the engineered probiotic EcN. The results reveal that SdiA acts as a global transcription factor in EcN, regulating NADPH metabolism of the (*S*)-equol-producing EcN-eq strain in the intestinal environment. The complex of C10-HSL with SdiA was shown to reduce the intracellular NADPH/NADP^+^ ratio by negatively regulating the expression of *zwf* and *gnd*. On this basis, the *sdiA* of EcN-eq was replaced with *sdiA_K12*, which has a low affinity for C10-HSL, resulting in the construction of an engineered EcN probiotic that efficiently produces (*S*)-equol in the intestinal environment. Considering the crucial role of NADPH as a cofactor in redox reactions, these findings offer valuable insights for engineering probiotics tailored to the gut environment. However, it is worth noting that this study was conducted in vitro; thus, in vivo experiments in an intestinal environment should be further implemented to confirm these findings.

## Figures and Tables

**Table 1:** Strains used in this study.

Strains	Relevant Properties	Source
*E. coli* DH5α (collection no.: DSM 6897)	F^−^, φ80d *lacZΔ*M15, Δ*(lacZYA-argF)*U169, *recA*1, *endA*1, *hsdR*17(rk^−^, mk^+^), *phoA*, *supE*44λ^−^, *thi*^−^, *gyrA*96, *relA*1	Invitrogen (Invitrogen, Carlsbad, CA, USA)
EcN (collection no.: DSM 115365)	Wild-type *E. coli* Nissle 1917	Lab stock
EcN-eq	EcN, *malK*::P*_nar_-dznr-*P*_nar_-ddrc*-P*_nar_-dhdr*-P*_nar_-thdr*, *exo/cea*::P*_nar_-bglF-*P*_nar_-bglB*, Δ*ptsG*::*Kan^R^*	This study
EcN-eq Δ*decR*	EcN-eq, Δ*decR*	This study
EcN-eq Δ*HW372_01960*	EcN-eq, Δ*HW372_01960*	This study
EcN-eq Δ*yhjC*	EcN-eq, Δ*yhjC*	This study
EcN-eq Δ*HW372_03545*	EcN-eq, Δ*HW372_03545*	This study
EcN-eq Δ*sdiA*	EcN-eq, Δ*sdiA*	This study
EcN-eq Δ*yhaJ*	EcN-eq, Δ*yhaJ*	This study
EcN-eq pETM6-P*_nar_*-*decR*	EcN-eq carrying pETM6-P*_nar_*-*decR*	This study
EcN-eq pETM6-P*_nar_*-*HW372_01960*	EcN-eq carrying pETM6-P*_nar_*-*HW372_01960*	This study
EcN-eq pETM6-P*_nar_*-*yhjC*	EcN-eq carrying pETM6-P*_nar_*-*yhjC*	This study
EcN-eq pETM6-P*_nar_*-*HW372_03545*	EcN-eq carrying pETM6-P*_nar_*-*HW372_03545*	This study
EcN-eq pETM6-P*_nar_*-*sdiA*	EcN-eq carrying pETM6-P*_nar_*-*sdiA*	This study
EcN-eq pETM6-P*_nar_*-*yhaJ*	EcN-eq carrying pETM6-P*_nar_*-*yhaJ*	This study
EcN-eq Δ*sdiA*::*sdiA_K12*	EcN-eq, Δ*sdiA*::*sdiA_K12*	This study

**Table 2:** Differential gene expression analysis of the EcN-eq strain in medium without/with the addition of SSF5.

Gene	Annotation	log_2_ (Fold Change)
Transporters		
*yagG*	sugar transporter	−2.9905
*ydcS*	polyamine transporter	−2.2575
*fetB*	iron-export ABC-transporter ATPase	2.5437
*gatA*	galactitol PTS	1.5344
Pentose phosphate pathway		
*zwf*	glucose-6-phosphate 1-dehydrogenase	−1.8321
*gnd*	6-phosphogluconate dehydrogenase	−2.6247
Acetate, anaplerotic, and other gluconeogenic pathways		
*poxB*	6-phosphofructokinase	2.7182
*acs*	acetyl-CoA synthetase	1.5124
*pfkA*	6-phosphofructokinase I	2.1273
*gpmM*	2,3-bisphosphoglycerate-independent phosphoglycerate mutase	1.5162
Stress-response pathway		
*rsfA*	ribosome-silencing factor	1.7628
*yfcV*	stress-response fimbriae	2.2365
*ypjA*	stress-response adhesin	1.9634
Cell to cell interaction		
*hlyE*	hemolysin E	2.6343
*ypfA*	adhesion-like autotransporter	1.7762
*fimA*	major type-I fimbrin	3.7689
*yqjH*	siderophore interaction protein	2.1547
Transcriptional regulator		
*decR*	AsnC-family transcriptional regulator	−1.9354
*ascG*	transcriptional regulator	3.2329
*yhjC*	LysR-family transcriptional regulator	2.7514
*frvR*	transcriptional regulator	−2.1672
*sdiA*	LuxI-family transcriptional regulator	3.4855
*yhaJ*	transcriptional regulator	2.7841

## Data Availability

Data are available on request from the authors.

## Supplementary Materials

The following supporting information can be downloaded at: https://www.mdpi.com/article/10.3390/antiox13030259/s1, Table S1. Nucleotide sequences of primers. Table S2. Vectors used in this study. Figure S1. Chiral HPLC analysis of daidzin metabolites converted by strain EcN-eq. (A). Reference standards of (*S*)-Equol and (*R*)-Equol. (B). HPLC spectrum displaying the reaction sample from strain EcN-eq. Figure S2. Densitometric semi-quantifications of the SDS-PAGE in Figure 2C. Experiments in this study were conducted in triplicate, and error bars signify standard deviation (SD) with a 95% confidence interval (CI). Figure S3. Sequence alignment of *E. coli K12*_SdiA and *EcN*_SdiA.
