# Supplementary material for: Deciphering the Crucial Roles of the Quorum-Sensing Transcription Factor SdiA in NADPH Metabolism and (S)-Equol Production in Escherichia coli Nissle 1917
Source: Antioxidants (Basel). 2024 Feb 20;13(3):259. doi: 10.3390/antiox13030259 (PMC10967497; doi:10.3390/antiox13030259)
Supplement: Supplementary file 1 [file antioxidants-13-00259-s001.zip › antioxidants-2851669-supplementary.pdf]

Deciphering the Crucial Roles of the Quorum-Sensing Transcription Factor SdiA in NADPH Metabolism and (*S*)-Equol Production in *Escherichia coli* Nissle 1917

Zhe Wang [1,2], Yiqiang Dai [1,2], Fidelis Azi [3], Mingsheng Dong [1,*] and Xiudong Xia [1,2,4,5,*]

[1] College of Food Science and Technology, Nanjing Agricultural University, Nanjing 210095, China; wangzhe@stu.njau.edu.cn (Z.W.); 2021208005@stu.njau.edu.cn (Y.D.)
[2] Institute of Agro-Product Processing, Jiangsu Academy of Agricultural Sciences, Nanjing 210014, China
[3] Department of Chemical Engineering, Guangdong Technion-Israel Institute of Technology, Shantou 515063, China; fidelis.azi@gtiit.edu.cn
[4] Jiangsu Key Laboratory for Food Quality and Safety-State Key Laboratory Cultivation Base, Ministry of Science and Technology, Nanjing 210014, China
[5] School of Food and Biological Engineering, Jiangsu University, Zhenjiang 212013, China
* Correspondence: dongms@njau.edu.cn (M.D.); 20140034@jaas.ac.cn (X.X.)

Supplementary Tables

Table S1. Nucleotide sequences of primers. Primer sequence utilized for homologous recombination is underlined.

**Oligonucleotides**	**Sequences, 5'-3'**
Pf_Pnar	GAAGGAGATATACATATGGCAGATCTCAATTGG
Pr_Pnar	ATGTATATCTCCTTCTTAAAGTTAAACAAATTCTTTAAGGGCATTATACCG C
Pf_*malEK* (up)	GCGGTCAGCATAATCATTCACC
Pr_*malEK* (up)	GGCGTCGACCCTAGGGAGACTGCTGCCGAAAGAGTCT
Pf_*malEK* (down)	CCTAGGGTCGACGCCCCCTGCTGTTCAAAACGTTTTG
Pr_*malEK* (down)	TCCGGTTACGGTAGGCAAC
Pf_*exo/cea* (up)	GCTCCCATATCCCAGAACTG
Pr_*exo/cea* (up)	GTCGACCCTAGGGCGGTCAGATTGAGTTCACCG
Pf_*exo/cea* (down)	ACCCCTAGGGTCGACCGGCATGGTCCCGGAAAACGGTA
Pr_*exo/cea* (down)	CCGCAATCATTTACGTTATCC
Pf_bglF	ATGGAAACGGAGTTAGCCAGAAAAAT
Pr_bglF	TTAGCGAATGATGGATAACAGCG
Pf_bglB	ATGAAAGCATTTCCAGAAACATTTCTT
Pr_bglB	TTAAGGTGCTTTAATGGTTATTTTTTTAATGACAG
Pf_ptsG (up)	CGTTATGTCCCCCTGGATC
Pr_ptsG (up)	CCTGAGTATGGGTGCTTTTT

Pf_ ptsG (down)	GAGTATGGGTGCTTTTTTGGCAGAAGCAGGCGGTT
Pr_ ptsG (down)	CTACCGGGTTCTGGTAAGC
Pf_ decR (up)	ATCCACCAGCGTCAGCAC
Pr_ decR (up)	CTTCCAGCTTCGCGATGGA
Pf_ decR (down)	TCGCGAAGCTGGAAGTCATGTCGGCAACCTGGAC
Pr_ decR (down)	ATGTTAGATAAAATTGACCGTAAGC
Pf_HW372_01960 (up)	GGCTCCCACTGTGAAATCGT
Pr_HW372_01960 (up)	GGTTGATCGCGTCTTTAATATCG
Pf_HW372_01960 (down)	AAGACGCGATCAACCGTGATTTCCAGGGGCAAGAT
Pr_HW372_01960 (down)	GCGGAAGTTGCGAGTAAAGC
Pf_ yhjC (up)	TGACCGATTGTTGTTCACAACG
Pr_ yhjC (up)	CTCCACCTGAATACGTTAAAAAGAC
Pf_ yhjC (down)	ACCCAGGTGCGGTGTATAAC
Pr_ yhjC (down)	ACTGGCTAACAACAAGAAGTGTTTCGCGCCAGATAC
Pf_HW372_03545 (up)	CGCAAGCTGGCAGAACTT
Pr_HW372_03545 (up)	ATTTATCTTCCGGTAGAGGTCGC
Pf_HW372_03545 (down)	CTACCGGAAGATAAATGCTACATTAATGAGCATCGTGAAG
Pr_HW372_03545 (down)	GCGCCATCATGATCAGAACATC
Pf_sdiA (up)	ACGTCGTTTGTCTGGCGG
Pr_sdiA (up)	ATAGTAAACCGCAACGCCCC
Pf_sdiA (down)	GTTGCGGTTTACTATGCAGCTGGAGTACGATTACTATTCG

Pr_sdiA (down)	GCTGATGTCTTACCTTCCGCC
Pf_yhaJ (up)	ACAACATAATCAGGTCGCGTC
Pr_yhaJ (up)	CACATTCGTTTGCAAAGGAAGG
Pf_yhaJ (down)	TGAGAACGAAATGGCCTTCCGCACTTAGCTACACC
Pr_yhaJ (down)	CCACGATATTTCACGCGCG
Pf_addsgRNA	GTTCCGTTTATCCGGGCAAAACTAGTATTATACCTAGGACTGAGC
Pr_malEK-sgRNA	TCTGCTCGACAAACCCTTCGTTTTAGAGCTAGAAATAGCAAGTT
Pr_exo/cea-sgRNA	ACTCCATACCCTCCCCAACGTTTTAGAGCTAGAAATAGCAAGT
Pr_HW372_01960-sgRNA	TGTGTTTGGAGATGTTCAGGTTTTAGAGCTAGAAATAGCAAGT
Pr_yhjC-sgRNA	GCAGTTGTTCATCAAAGTCGTTTTAGAGCTAGAAATAGCAAGT
Pr_HW372_03545-sgRNA	GCGCGAGTCAGTTAACGCCTTTTTAGAGCTAGAAATAGCAAGT
Pr_sdiA-sgRNA	TTCATGGTAGACCTCTTCTGTTTTAGAGCTAGAAATAGCAAGT
Pr_yhaJ -sgRNA	ATGGATGCGATCGATCGCCGTTTTAGAGCTAGAAATAGCAAGT
Pr_ ptsG-sgRNA	GTATCCGTACTGCCTATCGAGTTTTAGAGCTAGAAATAGCAAGT
Pr_decR -sgRNA	GTCTGGTAAACAGTGTACCGTTTTAGAGCTAGAAATAGCAAGT
F_16S (qPCR)	GTTAAGTCCCGCAACGAGCGCAA
R_q16S (qPCR)	CTTTATGAGGTCCGCTTGCTCTC
F_zwf (qPCR)	CCAAGCTGGATCTGAGCTATTC
R_zwf (qPCR)	CCACTTCATCACGACGTACAA
F_gnd (qPCR)	GAACCGCTGTCGCTGATTA
R_gnd (qPCR)	GGGCCAGAGAGAACTTTAGATG

Table S2. Vectors used in this study.

Vectors	Relevant properties	Reference
pETM6	pBR322 ori, AmpR, T7 promote	Addgene #49795
pEcCas	*Cas9* under *cas* promoter, gRNA-*pMB1* under *rha* promoter, λ-red under *araB* promoter, pSC101 ori, *KanR*	Addgene #73227
pEcgRNA	sgRNA under J23119 promoter, pMB1 ori, *aadA*	Addgene#166581
pETM6-P_{nar}	pBR322 ori, AmpR, *nar* promote	This study
pUC57-*malEK*	*malEK* homology arms, pBR322 ori	This study.
pUC57-*exo/cea*	*exo/cea* homology arms, pBR322 ori	This study.
pUC57-*ptsG*	*ptsG* homology arms, pBR322 ori	This study.
pUC57-*yhaJ*	*yhaJ* homology arms, pBR322 ori	This study.
pUC57-*decR*	*decR* homology arms, pBR322 ori	This study
pUC57-*HW372_01960*	*HW372_01960* homology arms, pBR322 ori	This study.
pUC57-*yhjC*	*yhjC* homology arms, pBR322 ori	This study.
pUC57-*HW372_03545*	*HW372_03545* homology arms, pBR322 ori	This study.
pUC57-*sdiA*	*sdiA* homology arms, pBR322 ori	This study.
pUC57-*exo/cea*-P_{nar}-*bglF*-P_{nar}-*bglB*	pUC57-*exo/cea* carrying -P_{nar}-*bglF*-P_{nar}-*bglB*	This study.
pUC57-*malEK*-P_{nar}-*dznr*-P_{nar}-*ddrc*-P_{nar}-*dhdr*-P_{nar}-*thdr*	pUC57-*malEK* carrying P_{nar}-*dznr*-P_{nar}-*ddrc*-P_{nar}-*dhdr*-P_{nar}-*thdr*	This study.
pEcgRNA-*ptsG*	Derived from pEcgRNA, target *ptsG* in EcN	This study.
pEcgRNA-*malEK*	Derived from pEcgRNA, target *malEK* in EcN	This study.
pEcgRNA-*exo/cea*	Derived from pEcgRNA, target *exo/cea* in EcN	This study.
pEcgRNA-*yhaJ*	Derived from pEcgRNA, target *yhaJ* in EcN	This study.
pEcgRNA-*decR*	Derived from pEcgRNA, target *decR* in EcN	This study.
pEcgRNA-*HW372_01960*	Derived from pEcgRNA, target *HW372_01960* in EcN	This study.
pEcgRNA-*yhjC*	Derived from pEcgRNA, target *yhjC* in EcN	This study.
pEcgRNA7-*HW372_03545*	Derived from pEcgRNA, target *HW372_03545* in EcN	This study.
pEcgRNA-*sdiA*	Derived from pEcgRNA, target *sdiA* in EcN	This study.
pETM6-P_{nar}-*yhaJ*	pETM6-P_{nar} harboring *yhaJ*	This study.
pETM6-P_{nar}-*decR*	pETM6-P_{nar} harboring *decR*	This study.
pETM6-P_{nar}-*HW372_01960*	pETM6-P_{nar} harboring *HW372_01960*	This study.
pETM6-P_{nar}-*yhjC*	pETM6-P_{nar} harboring *yhjC*	This study.
pETM6-P_{nar}-*HW372_03545*	pETM6-P_{nar} harboring *HW372_03545*	This study.
pETM6-P_{nar}-*sdiA*	pETM6-P_{nar} harboring *sdiA*	This study.

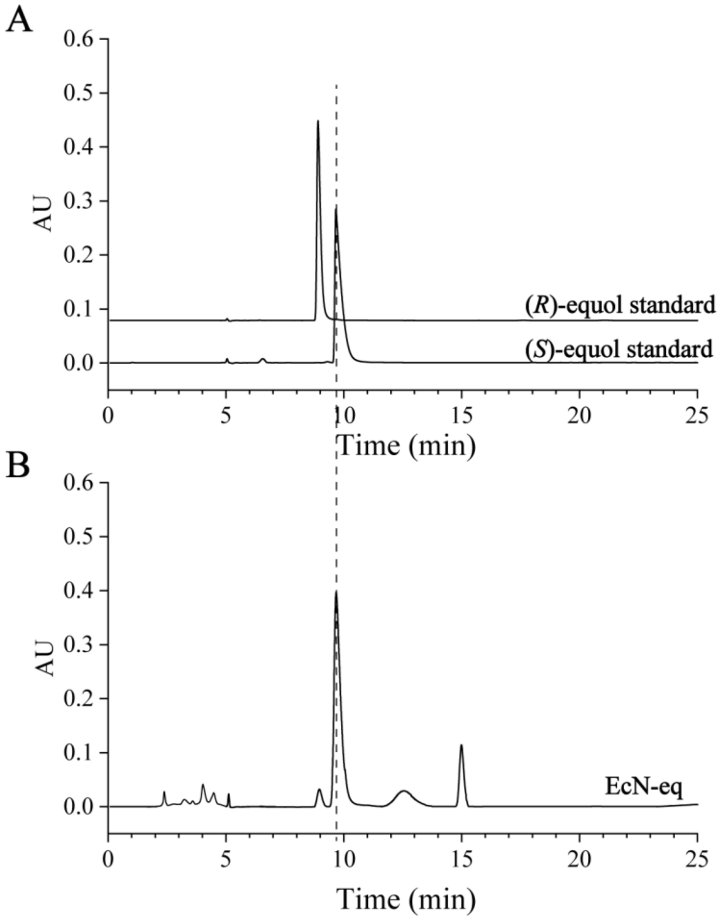

Fig. S1. Chiral HPLC analysis of daidzin metabolites converted by strain EcN-eq. (A). Reference standards of (*S*)-Equol and (*R*)-Equol. (B). HPLC spectrum displaying the reaction sample from strain EcN-eq.

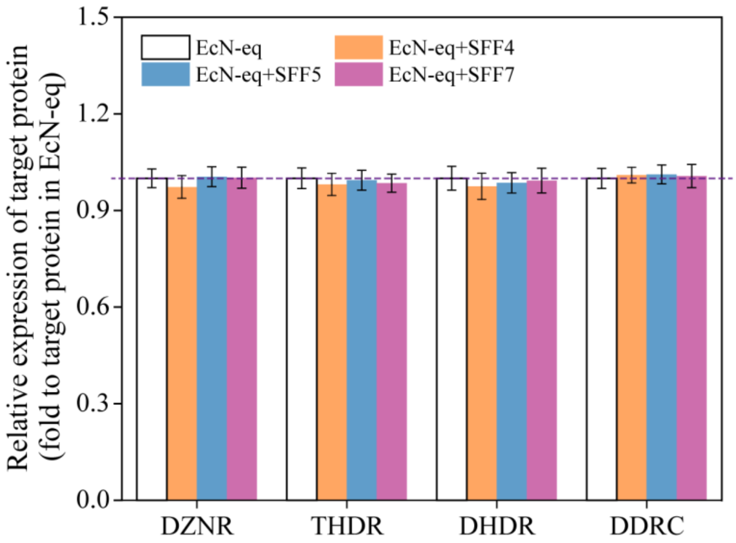

Fig. S2. Densitometric semi-quantifications of the SDS-PAGE in Fig. 2C. Experiments in this study were conducted in triplicate, and error bars signify standard deviation (SD) with a 95% confidence interval (CI).

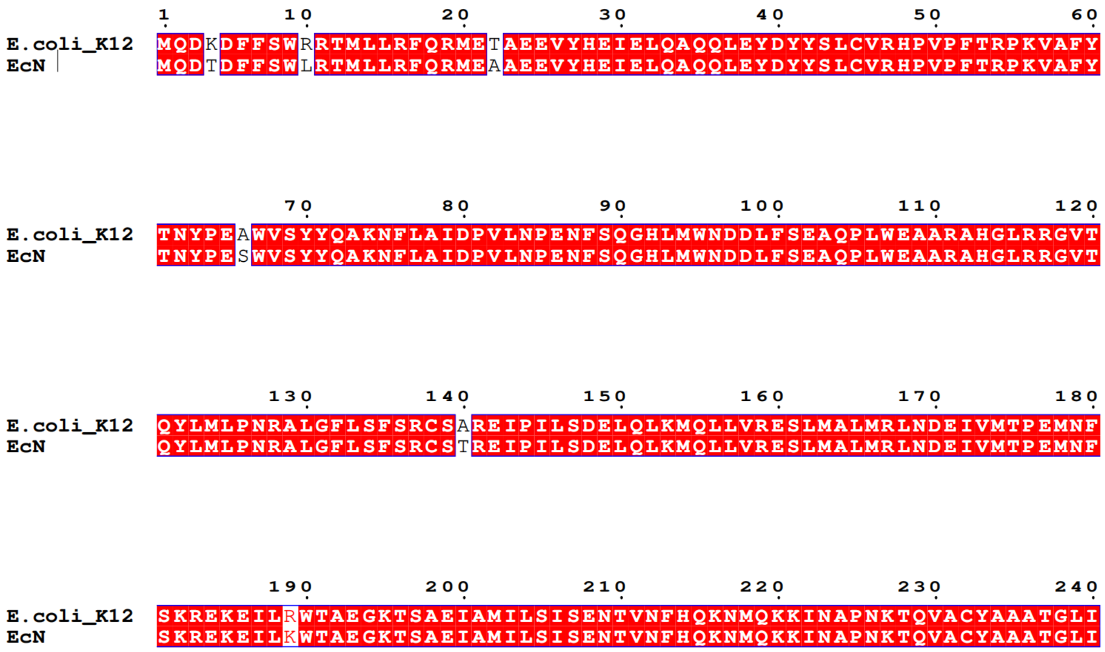

Fig. S3. Sequence alignment of *E. coli K12*_SdiA and *EcN*_SdiA. The red background box indicates identical sequence and red letters indicate diverse sequence.
